# Case Report: Response to Immunotherapy, Can Radiotherapy Be a Troublemaker?

**DOI:** 10.3389/fimmu.2021.745146

**Published:** 2021-11-04

**Authors:** Patricia Martin-Romano, Julien Adam, Jean-Yves Scoazec, Sébastien Gouy, Antonin Levy, Capucine Baldini, Stéphane Champiat, Jean-Charles Soria, Christophe Massard, Aurélien Marabelle, Eric Deutsch, Antoine Hollebecque

**Affiliations:** ^1^ Early Drug Development Department (DITEP), Gustave Roussy Cancer Campus, Villejuif, France; ^2^ Department of Pathology, Gustave Roussy Cancer Campus, Villejuif, France; ^3^ Department of Surgery, Gustave Roussy Cancer Campus, Villejuif, France; ^4^ Department of Radiation Oncology, Gustave Roussy Cancer Campus, Villejuif, France; ^5^ Institut National de la Santé et de la Recherche Médicale (INSERM), Gustave Roussy Cancer Campus, Villejuif, France; ^6^ Université Paris Sud, Université Paris-Saclay, Le Kremlin-Bicêtre, France

**Keywords:** immunotherapy, radiation therapy, tumor response, immune microenvironment, anal squamous cell carcinoma

## Abstract

Immunotherapy has dramatically changed the treatment landscape for several tumor types. However, the impact of previous radiotherapy (RT) on response to immunotherapy is still unknown. We report the case of a 58-year-old female diagnosed with a squamous anal cell carcinoma previously treated with RT and having a dissociated response to anti-PD1 agent. An extensive analysis of the immune contexture performed on the tissue collected from both previously RT-treated and RT-untreated lesions confirmed differences on immune microenvironment, highlighting the potential impact of radiotherapy on the immune response.

## Introduction

With an estimate of 8,580 new cases (2:1 women over men) in 2018 in the USA, anal cancer accounts for approximately 2.7% of all digestive system tumors (1). Although anal cancer is considered a rare disease, the number of patients diagnosed has increased in recent decades, especially squamous cell carcinomas (SCC), the most frequent histological subtype (2). Despite the fact that 90% of SCC anal cancer cases are caused by human papillomavirus (HPV) infection, little is known about its etiology.

Approximately 32% of patients are diagnosed with metastatic nodal locoregional disease (3). The standard upfront approach for these patients is definitive radiation (RT) with concurrent daily capecitabine as an oral prodrug of 5-fluorouracil (5-FU) and mitomycin, with 5-year survival estimates of approximately 62% (4). Unfortunately, more than 30% of patients present a relapse of the disease and there are no standard recommendations for this subgroup of patients (4). Several clinical trials have assessed cisplatin-based regimens, and more recently, a combination of docetaxel to cisplatin and 5-FU (5). Immunotherapy (IT) has been tested in two clinical trials evaluating anti-PD-1 efficacy in anal cancer patients who have progressed to standard therapy. Both studies showed promising results including a response rate of 20% and 24%, respectively (6, 7). Notwithstanding, both studies were nonrandomized clinical trials and included less than 50 patients. The impact of prior radiotherapy on tumor response to anti-PD1 remains unknown.

**Figure 1:**
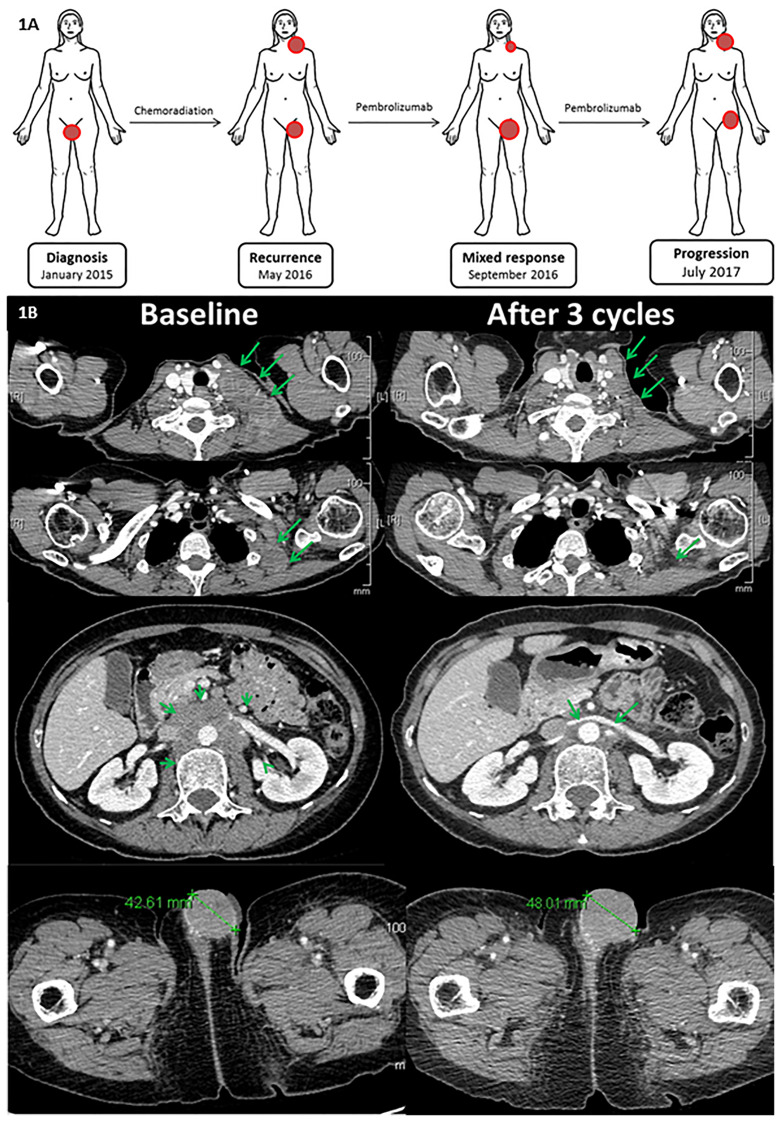
**(A)** Patient’s evolution including diagnosis, disease recurrence, mixed response during pembrolizumab, and disease progression to pembrolizumab. **(B)** Radiological assessment baseline and after three cycles (after 18 weeks): patient presented supraclavicular, mediastinal, lombo-aortic, inguinal lymph nodes, and a vulvar metastasis before treatment. After 18 weeks/three cycles of treatment, a partial response was observed. However, in the previously irradiated field, tumor continued to grow up.

We present the case of a 58-year-old female diagnosed with an anal SCC previously treated with RT and presenting a dissociated response to anti-PD-1 agent (pembrolizumab). An extensive analysis of the immune contexture performed on the tissue collected from both previously RT-treated and RT-untreated lesion highlighted the immunosuppressive impact of radiotherapy within the tumor microenvironment (**Figure 1A**).

## Case Description

In January 2015, a 58-year-old female was diagnosed with an anal squamous cell carcinoma (SCC) with left iliac and inguinal and para-aortic metastatic lymph nodes (cT2N2M1). Pathology report confirmed HPV infection within the tumor by p16 overexpression. Between February and March 2015, she was treated by intensity-modulated radiotherapy (IMRT) with concomitant chemotherapy (CT) by 5-fluorouracil and mitomycine C. Radiation dose administered was 45 Gray (Gy) in 25 fractions to a pelvic radiation field including the primary tumor rectal, inguinal, external, and internal iliac and presacral regions with an additional boost of 24 Gy in 12 fractions in both primary tumor and nodal areas. The total radiation dose given was 69 Gy. Patient presented an initial partial response 1 month after chemoradiation was completed.

In June 2015, she presented multinodal disease relapse within the retroperitoneal (interaortocaval, left para-aortic), thoracic (left axillary and mediastinal), and cervical (left internal jugular, supraclavicular) areas. The patient received a first-line metastatic combination of 5-FU and cetuximab, but she progressed within 2 months at the first evaluation. Patient was subsequently treated by a second line of chemotherapy by carboplatine and taxol with initial disease stability but progression by increase of metastatic lymph nodes within 4 months. In March 2016, she underwent a third line of systemic therapy by Navelbine that was stopped due to severe gastrointestinal adverse events (nausea and diarrhea).

In May 2016, patient was referred to Gustave Roussy Cancer Campus. Patient presented a good Eastern Cooperative Oncology Group performance status (PS = 1). Patient presented 1.5 × 2 cm supraclavicular and cervical lymph nodes and an indurated vulvar lesion on the left side of approximately 2 × 3 cm. Gustave Roussy Immune Score was classified as low risk (GRIm-Score = 0) (8). Computed tomography (CT) scan showed metastatic disease at multiple lymph nodes and both lungs (**Figure 1B**). Baseline target lesions (TL) were a subcarinal lymph node (25 mm), retrocaval lymph node (15 mm), superior right lobe lesion (10 mm), and left vulvar mass (41 mm). Nontarget lesions (NTL) were lymph nodes left axillary, left supraclavicular, mediastinal, and retroperitonal lymph nodes, along with lung micronodules. She was treated by an anti-PD-1 Immune Checkpoint Targeted Monoclonal Antibody (Pembrolizumab).

First cycle of pembrolizumab was administered on the 5th of July 2016. Patient underwent the first three cycles of anti-PD-1 agent without any immune-related adverse events. After one cycle, physical examination showed a decrease in the supraclavicular and cervical lymphadenopathies along with an increase of the vulvar mass. On 6th September, patient underwent her first evaluation CT scan. The radiological exam showed a mixed response with an increase of the left vulvar mass with a decrease in size of the rest of the target and nontarget lesions. Indeed, the CT scan showed a −42% decrease of TL including subcarinal lymph node (15 mm), retrocaval lymph node (14 mm), and superior right lobe lesion (0 mm) and an increase of +22% in the vulvar mass (50 mm) (**Figure 1B**). The CT scan showed neither an increase of NTL nor the appearance of any other new lesions. Patient did not have any other symptoms and was clinically stable. Patient was offered an on-purpose double tumor biopsy to detect molecular alterations by the use of Comparative Genomic Hybridization Array (Agilent Technology, 180K), next-generation sequencing (Ion AmpliSeq™ Cancer Hotspot Panel v2), and whole-exome sequencing. On 13th September 2016, two biopsies were performed, one of the cervical lymph node [responsive lesion (RL)] and one of the vulvar mass [progressive lesion (PL)]. Biopsy from the RL showed 30% cellularity along with mutations in FBXW7 (R479Q), loss of ATM, PRKCA, GRB2 amplification, and a tumor mutational burden (TMB) of 4.3 Mut/Mb. Biopsy from the PL displayed 60% cellularity and mutations in FBXW7 (R479Q, 39%), TSC2 (28%), KMT2D, KMT2C, MLL2, and a TMB of 5.02 Mut/Mb.

**Figure 2:**
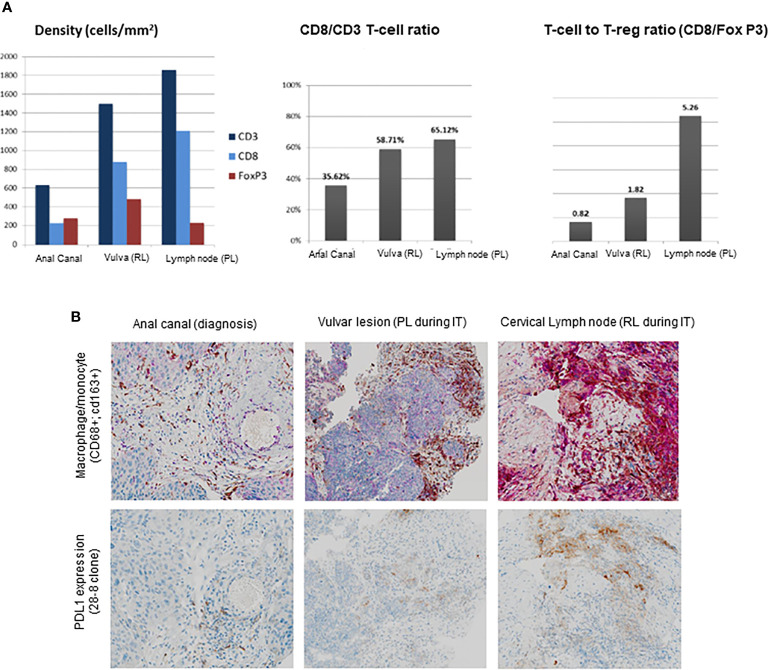
**(A)** Population of T cells in anal canal (baseline), vulvar lesion (PL), and cervical lymph node (RL). **(B)** Macrophage/monocyte presence **(A)** and PD-L1 expression **(B)** were evaluated at diagnosis in the anal canal (primary tumor) and during treatment with anti-PD-1 agent in the vulvar lesion (progressive lesion) and in the cervical lymph node (responsive lesion).

Lymphocyte T-cell populations as well as PD-L1 expression were assessed and compared between anal canal (baseline), vulvar lesion (PL), and cervical lymph node (RL) through immunohistochemistry and immunofluorescent staining. Population of T cells CD3(+), CD8(+), and CD4(+)CD25(+)FOXP3(+), CD8/CD3, and CD8/FOXP3 ratios in the three settings are represented on **Figure 2A**. CD8/CD3 T-cell ratio was 35.6 in the anal canal, 58.7 in the PL, and 65.1 in the RL. CD8/FOXP3 T cell ratio was <1% in the anal canal, 1.82 in the PL, and 50.26 in the RL (**Figure 2A**). Macrophage presence was evaluated by the expression of CD68 and CD163 by immunohistochemistry showing low expression in anal canal, moderate expression in the PL, and high expression in the RL. PD-L1 expression was found to be low in the anal canal, moderate in the PL, and high in the RL (**Figure 2B**). Transforming growth factor β (TGF-β) could not be evaluated.

Treatment with anti-PD1 was continued for two more cycles. On 11th November 2016, CT scan showed a complete response of the subcarinal lymph node (0 mm), retrocaval lymph node (0 mm), and superior right lobe lesion (0 mm), as well as 20% increase of the vulvar mass (62 mm). NTL were classified as stable disease. Patient was discussed by the multidisciplinary panel who decided to perform a partial left vulvectomy with postoperative radiation. On 14th December 2016, a surgery consisting of a partial left vulvectomy was performed. Pathologic report showed an ulcerated infiltrating nonkeratinizing epidermoid carcinoma measuring 10 × 7 cm with carcinomatous emboli and free surgical margins. Given the clinical benefit of the disease, anti-PD1 therapy was resumed in January 2017. A new CT scan performed on 16th May 2017 displayed an increase of NTL (left axillary, left supraclavicular, retroperitoneal, and right inguinal) as well as the appearance subcutaneous nodule on the left thigh root (29 mm). Given the clinical benefit and the absence of new target lesions, treatment was continued although a biopsy of the progressing inguinal lymph node was done on 11th July 2018. Anatomopathological report confirmed the presence of a mutation of pathogenic variant of FBXW7, TCC2, MAP3K1, ESR1, and PALPB2. On 18th July 2017, a new CT scan showed the progression of the disease with an increase of NTL including left supraclavicular lymph node (35 × 39 *vs*. 16 × 19 mm), left axillary lymph node (28 × 33 *vs*. 11 × 15 mm), right inguinal lymph node (33 × 40 *vs*. 27 × 15 mm), and an increase of the subcutaneous lesion infiltrating the thigh root (35 mm). Treatment was then discontinued on 18th July 2017 for progression.

## Discussion

We present the case of a 58-year-old female diagnosed with a squamous anal cell carcinoma initially treated with RT. After presenting a disease progression, she was treated with an anti-PD-1 immune checkpoint inhibitor. The first radiological evaluation showed a dissociated response with an important progression of the lesion included in the initial radiation field and a partial response of nonirradiated lesions. An extensive analysis of the immune contexture performed on the tissue collected from both responsive and progressive lesions displays the potential impact that RT may have on subsequent immune responses. We hypothesized that the previously irradiated and progressive lesion had an immunosuppressive microenvironment due to earlier RT.

Chemotherapy and radiation therapy are the standard treatments to induce cancer cell death. On the one hand, CT is responsible of evolving oncogenic mutations and clonal selection (9). On the other hand, RT is also responsible for many biological effects in both tumor cells and tumor microenvironment, including immune modulation, tumor vasculature, and stroma. These biological effects involve the induction of an immunosuppressive *milieu* through the mobilization of locally suppressive immune cells as myeloid-derived suppressor cells (MDSC), tumor-associated macrophages (TAM) with a protumorigenic phenotype M2-like and FOXP3 regulatory T cell, as well as cytokines triggering immunosuppressive microenvironment such as transforming growth factor β (TGF-β) (10, 11). Primary tumor (anal canal), PL, and RL from our patient were extensively studied. Particularly, immune cell subpopulation assessment showed a higher rate of FOXP3 Tregs in both anal canal and PL, compared with RL. Interestingly, FOXP3 Tregs were significantly superior in previously irradiated field, confirming the potential immunosuppressive role of radiation. Expression of PD-L1 has been correlated to responses to anti-PD-1 therapy in many tumor types, and so has been suggested to play a role as a predictive factor (6). A recently published study evaluating pembrolizumab in advanced pretreated SCC anal cancer patients showed that PD-L1 was expressed in 74% of these patients (6). Nevertheless, PD-L1 expression was not associated to immune-tumor response in patients. A different study assessing anti-PD1 agent nivolumab reported that patients responding to had a significantly superior PD-L1 expression when compared with nonresponding patients (40% *vs*. 10%, *p* = 0.0056) (7). Interestingly, expression of PD-L1, which is a well-known mechanism of adaptive immune resistance by the inhibition of antitumor T-cell responses, has also been correlated to a lower survival and worse prognosis in SCC anal cancer patients (12). Biopsies from our patient displayed an inflammatory infiltrate with a PD-L1 expression in the RL that significantly higher when compared with the PL, where PD-L1 expression was lower. Other factors, as for instance, mutational tumor burden had been suggested as predictive factor for response to immunotherapy in different cancer types (13). Pathologic results from our patient showed a similar low-intermediate TMB in both PL (5.02 Mut/Mb) and RL (4.3 Mut/Mb), suggesting mutational load would have not been a factor impacting immune response from our patient. HPV infection has also been linked to a higher expression of tumor-infiltrating lymphocytes and PD-1, suggesting to playing a crucial role in the immune and inflammatory responses in anal cancer (14). Research of predictive biomarkers of immune responses in SCC anal cancer patients should consider that the vast majority of SCC anal cancer patients have already received a definitive radiation therapy.

Tumor-infiltrating lymphocytes (CD8+ T cells) in SCC anal cancer patients have been found to relate to a response to anti-PD-1 checkpoint inhibitor nivolumab (7). In responding patients, an increase in CD8^+^ T-cell infiltration was observed when comparing baseline and ongoing anti-PD-1 treatment. Presence of tumor-infiltrating lymphocytes has also been reported as a favorable prognostic factor in SCC anal cancer patients (15). Prevalence of CD8^+^ T-cell population in our patient’s biopsies was superior in the RL compared with the PL. It could be implied that RT might be able to exert a late decrease of tumor-infiltrating lymphocytes a subset of T cells crucial in the immune response, although it should be considered hypothetical in the absence of a direct comparison of the immune microenvironment from our patient before and after RT. In order to better understand tumor microenvironment from a previously irradiated field, a deeper comprehension of the radiation late effects is needed in patients with anal cancer.

## Conclusions

Understanding the interactions between tumor cells and the tumor microenvironment before and after cancer therapy remains a real challenge. RT might exert important immunosuppressive effects in the TME. Additionally, the increasing use of immunotherapy raises many questions regarding the possibility of combining different therapeutic strategies and how to design these multimodal approaches. Repeated tumor biopsies help us not only to understand tumor microenvironment of a previously irradiated field but also mechanisms of both response and resistance to checkpoint inhibitors.

## Data Availability Statement

The data presented in the study are deposited in the Figshare repository, doi: 10.6084/m9.figshare.16803661.

## Ethics Statement

The studies involving human participants were reviewed and approved by CSET2940. The patients/participants provided their written informed consent to participate in this study.

## Author Contributions

AH, J-YS, JA, SC, AL, ED, and CB were the treating physicians of the patient and recollected all the medical history of the patient. PM-R, AH, J-YS, JA, SC, AC, and ED wrote the core of this article. J-YS and JA performed all the pathologic analysis. AM and J-CS helped in the reviewing and editing of the article. All authors contributed to the article and approved the submitted version.

## Conflict of Interest

All authors declare: Principal/sub-Investigator of Clinical Trials for Abbvie, Agios Pharmaceuticals, Amgen, Argen-X Bvba, Arno Therapeutics, Astex Pharmaceuticals, Astra Zeneca, Aveo, Bayer Healthcare Ag, Bbb Technologies Bv, Blueprint Medicines, Boehringer Ingelheim, Bristol Myers Squibb, Celgene Corporation, Chugai Pharmaceutical Co., Clovis Oncology, Daiichi Sankyo, Debiopharm S.A., Eisai, Eli Lilly, Exelixis, Forma, Gamamabs, Genentech, Inc., Glaxosmithkline, H3 Biomedicine, Inc, Hoffmann La Roche Ag, Innate Pharma, Iris Servier, Janssen Cilag, Kyowa Kirin Pharm. Dev., Inc., Loxo Oncology, Lytix Biopharma As, Menarini Ricerche, Merck Sharp & Dohme Chibret, Merrimack Pharmaceuticals, Merus, Millennium Pharmaceuticals, Nanobiotix, Nektar Therapeutics, Novartis Pharma, Octimet Oncology Nv, Oncoethix, Onyx Therapeutics, Orion Pharma, Oryzon Genomics, Pfizer, Pharma Mar, Pierre Fabre, Roche, Sanofi Aventis, Taiho Pharma, Tesaro, Inc, Xencor. Research Grants from Astrazeneca, BMS, Boehringer Ingelheim, Janssen Cilag, Merck, Novartis, Pfizer, Roche, Sanofi. Non-financial support (drug supplied) from Astrazeneca, Bayer, BMS, Boringher Ingelheim, Johnson & Johnson, Lilly, Merck, NH TherAGuiX, Pfizer, Roche.

Additionally, PM-R has received honoraria from Roche, AbilityPharma and Astrazeneca. CM has received honoraria from Astellas, Astra Zeneca, Bayer, BeiGene, BMS, Celgene, Debiopharm, Genentech, Ipsen, Janssen, Lilly, MSD, Novartis, Pfizer, Roche, Sanofi, Orion; SC has received honoraria from AstraZeneca, BMS, Janssen, MSD, Novartis and Roche. AH has received honoraria from Amgen, Spectrum Pharmaceuticals, Lilly. JC-S has received consultancy fees from AstraZeneca, Astex, Clovis, GSK, GamaMabs, Lilly, MSD, Mission Therapeutics, Merus, Pfizer, PharmaMar, Pierre Fabre, Roche/Genentech, Sanofi, Servier, Symphogen, and Takeda. He is a shareholder of Amgen and Gritstone. AM has received honoraria from GSK, AstraZeneca, Oncovir, Merck Serono, eTheRNA, Lytix pharma, Kyowa Kirin Pharma, Bayer, Novartis, BMS, Symphogen, Genmab, Amgen, Biothera, Nektar, Pfizer, Seattle Genetics, Flexus Bio, Roche/Genentech, OSE immunotherapeutics, Transgene, Gritstone, Merck (MSD), Cerenis, Innate pharma, Protagen, Partner Therapeutics, Servier. ED has received honoraria from BMS, Roche and MSD. CB has received honoraria from BMS, Roche, and MSD.

## Publisher’s Note

All claims expressed in this article are solely those of the authors and do not necessarily represent those of their affiliated organizations, or those of the publisher, the editors and the reviewers. Any product that may be evaluated in this article, or claim that may be made by its manufacturer, is not guaranteed or endorsed by the publisher.

## Abbreviations

SCC, squamous cell carcinomas; HPV, human papillomavirus; RT, radiation; 5-FU, 5-fluorouracil; IT, immunotherapy; IMRT, intensity-modulated radiotherapy; PS, performance status; GRIm-Score, Gustave Roussy Immune Score; CT, computed tomography; TL, target lesions; NTL, nontarget lesions; MDSC, myeloid-derived suppressor cells; TAM, tumor-associated macrophages; PL, progressive lesion; RL, responsive lesion.
